# Discrepancies between prokaryotes and eukaryotes need to be considered in soil DNA‐based studies

**DOI:** 10.1111/1462-2920.16019

**Published:** 2022-04-24

**Authors:** Enrique Lara, David Singer, Stefan Geisen

**Affiliations:** ^1^ Real Jardín Botánico‐CSIC, Plaza de Murillo 2 Madrid 28014 Spain; ^2^ UMR CNRS 6112 LPG‐BIAF Angers University, 2 Boulevard Lavoisier Angers 49045 France; ^3^ Laboratory of Nematology Wageningen University Wageningen 6700 AA The Netherlands

## Abstract

Metabarcoding approaches are exponentially increasing our understanding of soil biodiversity, with a major focus on the bacterial part of the microbiome. Part of the soil diversity are also eukaryotes that include fungi, algae, protists and Metazoa. Nowadays, soil eukaryotes are targeted with the same approaches developed for bacteria and archaea (prokaryotes). However, fundamental differences exist between domains. After providing a short historical overview of the developments of metabarcoding applied to environmental microbiology, we compile the most important differences between domains that prevent direct method transfers between prokaryotic and eukaryotic soil metabarcoding approaches, currently dominated by short‐read sequencing. These include the existence of divergent diversity concepts and the variations in eukaryotic morphology that affect sampling and DNA extraction. Furthermore, eukaryotes experienced much more variable evolutionary rates than prokaryotes, which prevent capturing the entire eukaryotic diversity in a soil with a single amplification protocol fit for short‐read sequencing. In the final part we focus on future potentials for optimization of eukaryotic metabarcoding that include superior possibility of functionally characterizing eukaryotes and to extend the current information obtained, such as by adding a real quantitative component. This review should optimize future metabarcoding approaches targeting soil eukaryotes and kickstart this promising research direction.

## Introduction

The use of molecular approaches to study soil microorganisms catalysed the immense development of microbial ecology. While soil bacterial diversity has received attention since the late 80s, studies on microbial eukaryotes (fungi, protists and micro‐algae, but also soil microfauna) have been lagging behind. Yet, they are currently gaining momentum, as large/global‐wide studies (Bates *et al*., 2013; Tedersoo *et al*., 2014; Mahé *et al*., 2017; Oliverio *et al*., 2020; Aslani *et al*., 2022) are generating immense amounts of data. The global understanding of diversity distribution and community assemblage rules (Aslani *et al*., 2022), as well as the discovery of novel environmental clades with high relevance for deep eukaryotic phylogeny (Burki *et al*., 2020), has considerably increased our understanding of soil eukaryotic diversity. Major breakthroughs were made, such as showing that eukaryotes in soils are more diverse than in aquatic systems (Singer *et al*., 2021), the most dominant eukaryotes are phagotrophs with small protists dominating (Oliverio *et al*., 2020) and that phototrophs are major contributors of the global carbon cycle (Jassey *et al*., 2022).

The precursory nature of ecological environmental studies focusing on prokaryotes has led to the fact that eukaryote‐focused environmental diversity studies use largely the same methodology. Nevertheless, the last 10 years saw the development of new curated genetic databases dedicated to eukaryotes (Guillou *et al*., 2012) and the expansion of existing ones (Quast *et al*., 2013). However, sampling designs, laboratory protocols, data analyses tools and concepts that are used by most soil ecologists interested in eukaryotes are still mostly based on – or even are identical to – the approaches used to study prokaryotes.

Our aim in this perspective is to highlight fundamental differences between prokaryotes and eukaryotes, and the resulting consequences for environmental metabarcoding studies. Indeed, the immense morphological diversity of eukaryotes as well as their fast and inhomogeneous evolutionary rates prevent the application of protocols for eukaryotic metabarcoding that were developed to study prokaryotes. Indeed, unified protocols for all eukaryotes can only provide partial images of the domain's diversity in soils. In addition to this comparative list of warnings, we highlight avenues to take to optimize the methodology to study soil eukaryotes.

## Historical context; why did the study of eukaryotes lag behind prokaryotic studies?

Environmental microbiology with a focus on an *in toto* nucleic acid extraction, the current standard to assess microbial biodiversity, has started to reveal the immense diversity of planktonic bacteria in the Sargasso Sea (Lane *et al*., 1985; Giovannoni *et al*., 1990). Similar approaches followed shortly afterwards for Archaea (DeLong, 1992). Protocols were developed at that time to extract environmental DNA from soil (Tsai and Olson, 1992). These approaches led to the discovery of many major lineages composed exclusively of uncultured organisms, called ‘environmental clades’ (for a review encompassing all domains of life, see López‐García and Moreira (2008)). While prokaryotic microbiology developed considerably, microbial eukaryotic environmental diversity remained unstudied for more than a decade. Casually, the first study aimed at characterizing changes in communities in artificial systems after virus‐induced lysis in filamentous cyanobacteria (van Hannen *et al*., 1999). Eukaryotes gained more attention in aquatic systems, and studies revealed novel eukaryotic clades in marine (López‐García *et al*., 2001; Moon‐van der Staay *et al*., 2001) and freshwater hyperacidic systems (Amaral‐Zettler *et al*., 2002). Early environmental molecular explorations of soil eukaryotic diversity were still hampered by the low sequencing depth provided by amplicon cloning/Sanger sequencing approaches used by then, which did not allow seeing beyond the over‐dominance of a few plant and fungal operational taxonomic units (OTUs). Most sequences of protists, fungi and animals could not be retrieved (Lesaulnier *et al*., 2008), thus overlooking most eukaryotic diversity. Taxon‐specific primers circumvented this problem and were applied to investigate certain eukaryotic groups such as among protists (Bass and Cavalier‐Smith, 2004; Lara *et al*., 2007). In the meanwhile, soil bacteria studies were flourishing (Borneman *et al*., 1996; Chelius and Triplett, 2001; Sessitsch *et al*., 2001; Zhou *et al*., 2002). Later, the development and application of high‐throughput sequencing (HTS) technologies allowed comprehensive insights into microbial diversity in soils, such as of bacteria (Roesch *et al*., 2007), leading eventually to a better understanding of the distribution of bacterial diversity across global ecosystems (Delgado‐Baquerizo *et al*., 2018).

These methodological developments also benefited eukaryotic diversity surveys, as they allow overcoming the obstacle represented by the few over‐dominant OTUs and make possible the retrieval of a more complete picture of the eukaryotic biodiversity in soils. Eventually, the development of HTS in the last decade saw the considerable development of large soil metabarcoding initiatives, including global diversity surveys (Bates *et al*., 2013; Oliverio *et al*., 2020; Aslani *et al*., 2022). All these eukaryote‐focused approaches are mainly transferred from prokaryotic examples, often even using the same samples taken for prokaryotes for which the sampling has been optimized. The question remains how well transferable these approaches are and how accurately these data reflect the true eukaryotic diversity found in soils.

## Two different diversity concepts

**Fig. 1:**
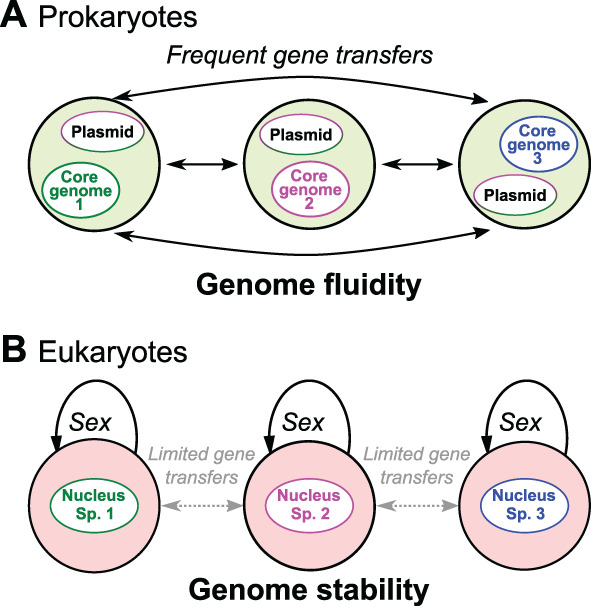
Differences between prokaryotic and eukaryotic diversity. While the first exchange functional genes frequently between taxa (A), eukaryotic genomes are much more stable and functions can be related to taxonomic affiliation (B).

Species (i.e. independent evolutionary units) are the basic unit of biological diversity. While this concept has largely been developed based on macroscopic and sexual eukaryotes such as plants and animals, species are more challenging to delimit in the asexual prokaryotes. Some researchers even doubt the meaningfulness of the notion of species in prokaryotes, where genes are commonly exchanged between organisms (Doolittle and Zhaxybayeva, 2009). Indeed, bacterial and archaeal genomes possess a relatively restricted number of specific genes (‘core genome’), while many functional genes related to adaptations to the environment are freely transferred horizontally through plasmid exchanges and conjugation (Paquola *et al*., 2018; Kloub *et al*., 2021) (Fig. 1). Diversity units are therefore constituted by these core genomes (Bobay, 2020) which also include relatively variable genes that can be used to differentiate between close related clinical strains (Bourdin *et al*., 2021) or to observe geographical isolation (Qin *et al*., 2022). Yet, prokaryotic environmental studies aim at a more complete overview of the prokaryotic diversity than the few taxa for which genomes can be compiled as metagenome‐assembled genomes (Howe *et al*., 2014). These studies still need to apply metabarcoding based on arbitrary criteria such as genetic distances between strains to delimit species, such as the 97% 16S rRNA gene identity ‘rule’ (Stackebrandt *et al*., 2002) or amplicon sequence variants (Callahan *et al*., 2017). The delimitation of independent evolutionary units still remains unclear in bacteria, therefore the link between OTUs and bacterial biological species is not obvious. An example is provided by the human pathogen *Salmonella flexneri*, which shares 99.9% similarity with *Escherichia coli* on the 16S rRNA gene (Fukushima *et al*., 2002), with even *E*.*coli* variants ranging from pathogenic to mutualistic (Dethlefsen *et al*., 2007). A recent study showed that more than hundred thousand *E*. *coli* and *Shigella* genomes varied in their pathogenicity depending on genomic features which are invisible at the 16S rRNA gene level (Abram *et al*., 2021).

In eukaryotes, biodiversity has been studied first on organisms that are easy to observe, that is, plants and animals, where genetic cohesion can essentially be characterized by sexual compatibility and population structure. These rules can also be applied to eukaryotic microbes, as most are considered sexual (Hofstatter and Lahr, 2019). Therefore, scientists interested in soil eukaryotes mostly benefit from the concept of species and all its developments (De Queiroz, 2007) as a benchmark for diversity. Genomes are much more stable in the eukaryotic world, and even if horizontal gene transfers have played a role in evolutionary histories, they remain extremely rare and would not bias diversity estimations (Van Etten and Bhattacharya, 2020) (Fig. 1). The term ‘core genome’ previously mentioned for bacteria does not apply here, as all genes are transmitted mostly within species, therefore, the OTUs obtained in environmental diversity studies are representative of the diversity. It must be noted that in microbial eukaryotes, organisms form temporal to permanent associations, most often composed by a consumer and a phototrophic organism (the consumer is then called ‘mixotroph’ by protistologists). Metabarcoding studies will retrieve sequences of both organisms, which we also consider as separate, like bacteria in a consortium.

**Fig. 2:**
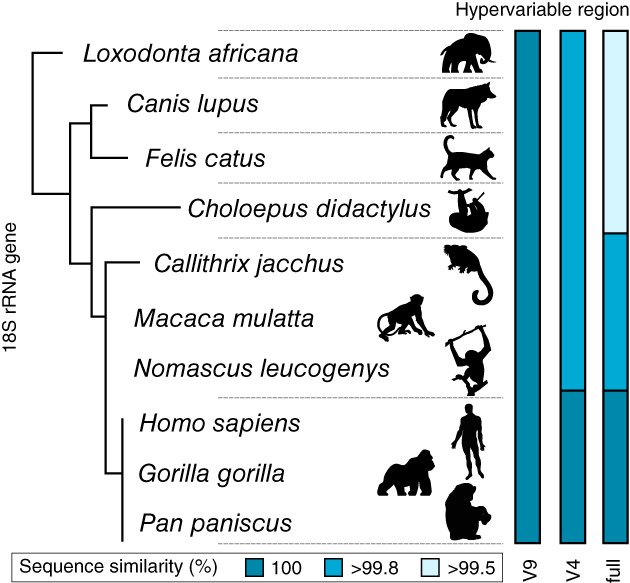
Phylogenetic tree illustrating percentages of genetic similarity between humans and different mammals based on some commonly used markers. v4 and v9 correspond to variable regions of the 18S rRNA gene.

Also in eukaryotes, the identity of species is not directly translatable from the commonly used 18S rRNA gene focused on in metabarcoding approaches. The reason is that this gene is simply not variable enough in most taxa. We illustrate this caveat in Fig. 2, showing that humans share exactly the same full 18S rRNA gene sequence with hominid primates (Fig. 2). Eukaryotic environmental metabarcoding targets even shorter parts of this gene, mostly the ‘hypervariable’ regions v4 and v9 of the 18S (Vaulot *et al*., 2022). Therefore, the reduced amount of phylogenetic information retrieved in current metabarcoding approaches would classify humans within the same taxonomic unit as elephants (Fig. 2). This vision would obfuscate a tremendous amount of diversity as the genetic information would be systematically pooled. This could lead to the fact that rare and potentially endangered species could be considered abundant if classified the same as some common species. For this reason, while 18S rRNA gene metabarcoding may provide a general overview of eukaryotic diversity in a soil, OTUs cannot be considered as equivalent to species, the basic unit of diversity. In order to allow species delimitation in eukaryotic metabarcoding studies by gaining taxonomic resolution, several variable genetic markers have been developed for the different groups and tested for specific resolution. The nuclear internal transcribed spacer (ITS), the mitochondrial cytochrome oxidase (COI) and the chloroplastic 23S rRNA in photosynthetic organisms are more variable than the 18S rRNA gene (Pawlowski *et al*., 2012) and are (or could be) used in group‐specific metabarcoding (see Section Potential for species‐level resolution in eukaryotic metabarcoding).

## Different morphologies change sampling protocols

**Fig. 3:**
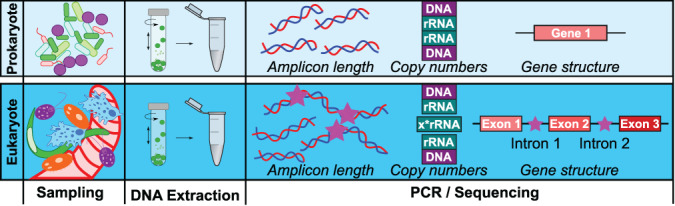
Schematic workflow comparison between prokaryotes and eukaryotes. Sampling and DNA extraction steps will be performed according to the targeted organisms. Fundamental differences between prokaryotes and eukaryotes (amplicon length, gene copy numbers and presence of intron in the eukaryote gene) can be observed in the PCR/Sequencing steps.

Eukaryotes are characterized by an immense morphological variation (in terms of size, shape and many other traits) that greatly exceeds that of bacteria and archaea (Fig. 3). These variations affect how sampling for metabarcoding experiments in soil needs to be conducted. As soil eukaryotes range from few micrometres (several flagellated and amoeboid protists) to the centimetre or even meter scale (earthworms, burrowing animals and fungal mycelia), sample size and distribution need to be increased compared to studies focusing on the purely microscopic prokaryotes (Jurburg *et al*., 2021; Potapov *et al*., 2022). Sampling for the smallest and most abundant eukaryotic consumers – nanoflagellates and ‘naked’ amoebae might be the same as for bacteria. These eukaryotic organisms are considered the main drivers of nutrient cycling in soils (Rønn *et al*., 2002). However, even larger unicells such as Arcellinida (testate amoebae) would then need further upscaling or possibly filtering for concentrating cells (Kosakyan *et al*., 2015). These microbial top predators can dominate microbial biomass in certain systems such as peat bogs (Jassey *et al*., 2013; Marcisz *et al*., 2014). Also, microscopic nematodes, the most abundant animals on Earth that can exceed the importance of protists for nutrient cycling in some soils (Coleman *et al*., 1984; Griffiths, 1990), cannot be sampled with the prokaryotic protocols as sample sizes exceeding 100 g of soil are needed for reliable diversity analyses (Wiesel *et al*., 2015). Due to the functional overlap of the key microbiome predators protists and nematodes, as well as other fauna that perform similar functions (Thakur and Geisen, 2019), a separation between metazoans and unicellular organisms would be artificial and provide a biased vision of eukaryotic functional diversity. Like for large protists, nematodes and certainly also rotifers are extracted from the soil matrix before molecular work (Kawanobe *et al*., 2021). In order to retrieve all microbial‐sized eukaryotic diversity in metabarcoding analyses, huge amounts of soil should be taken. This would increase dramatically complicate and increase costs of sampling, and setting up controlled experiments such as mesocosms. At the same time, spatial heterogeneity of smaller organisms would be shadowed by sampling large amounts. A solution may come from applying different sampling strategies that target different organisms size classes and pooling nucleic acids extractions together. Sampling protocols to be applied depend therefore on the research question (and mostly on the target taxa), but also on the type of soil investigated.

After sampling, nucleic acids need to be extracted in order to continue the flowchain in environmental metabarcoding. Some bacterial and archaeal taxa are recalcitrant to generic extraction protocols, as for instance the highly resistant spore‐forming bacteria (Dineen *et al*., 2010). In eukaryotes, differences between taxa exist also given their variability in morphology. In soil, most protists have the capacity to produce resistant dormant life stages or cysts (Ekelund and Rønn, 1994; Geisen *et al*., 2018). Some of these cysts are extremely resistant, and can withstand wet heat up to 50°C and dry heat to 120°C (Fenchel, 1987). This resistance is shown by successful resurrection of protist taxa after tenths of thousands of years from Arctic permafrost (Shmakova and Rivkina, 2015; Shmakova *et al*., 2016). The particularly recalcitrant cysts of *Acanthamoeba* spp. are also highly resistant to a variety of chemical agents (Turner *et al*., 2000). In turn, soil protist diversity includes highly fragile and network forming amoeboid organisms, which are abundant and widespread, such as foraminiferans (Holzmann *et al*., 2021) and variosean amoebae (Berney *et al*., 2015). These organisms, which can reach sizes of more than 1 mm, are disrupted when soil is ground or sieved, and their DNA can get lost. These differences make it impossible to assess eukaryotic diversity in an unbiased way using a single nucleic acid extraction approach. As it is mostly not feasible to combine many different protocols and it is also not always the goal to uncover the entire eukaryotic diversity, we propose to use a single extraction approach (Santos *et al*., 2017). Ideally, biases in extraction should be known to reliably discuss potential presence, absence and abundance of eukaryotic taxa present in a sample, and although certain trends are identifiable (Santos *et al*., 2017), taxon oriented‐research would be needed to adapt protocols to target eukaryotic groups.

## Consequences of rRNA evolutionary rates on eukaryotic PCR‐based surveys

After sampling and extracting nucleic acids, the amplification of DNA extractions for environmental DNA surveys is also a source of bias that differs between prokaryotes and eukaryotes. One of the premises of environmental metabarcoding surveys is that taxa are equally amplified in all clades, as the sequences of the designed primers are highly conserved through evolution. For prokaryotes, protocols have been developed to obtain an optimized coverage of the whole diversity using primers flanking the v3–v5 variable regions of the 16S rRNA gene, with a size that is suited for short, max 550 bp targeting Illumina sequencing. For instance, the primer pair 515f/806r 16S SSU rRNA gene has been designed to match perfectly with flanking regions of more than 90% of all bacterial and archaeal 16S barcoding regions (Walters *et al*., 2011), making these oligonucleotides excellent broad‐range primers to cover prokaryotes (Knight *et al*., 2018). A recent test (03/2022) performed on the SILVA database (Klindworth *et al*., 2012) still shows that, despite the upload of most recent data, these primers still accommodate over 86% of all bacterial and archaeal sequences.

In comparison to their bacterial counterparts, eukaryotic ribosomal 18S rRNA genes present profoundly more heterogeneity in the regions flanking the most variable barcoding regions. Indeed, eukaryotes are famous for their fast‐evolving ribosomal genes that changed at different paces between clades. As an illustration, diverging evolution paces between eukaryotic 18S rRNA genes caused artefacts that disrupted the topology of the first eukaryotic trees (‘long branch attraction’) and caused misinterpretations on the evolutionary history of the whole domain (Philippe and Germot, 2000). These 18S rRNA gene‐related differences still cause issues in metabarcoding studies. The first consequence is that a universal PCR protocol cannot be designed for the whole domain Eukarya, because of a lack of universally conserved regions for primer design. Therefore, eukaryotic environmental molecular diversity studies based on PCRs performed on the 18S rRNA gene are systematically biased against certain organisms (Vaulot *et al*., 2022). Some common and diverse soil groups, such as the protistan Amoebozoa and Heterolobosea (Discoba) usually have sequences that are too variable in even conserved regions to design perfectly matching primers (Vaulot *et al*., 2022). Unfortunately, primers do not only ‘eliminate’ certain large clades from metabarcoding data. Evolutionary paces vary within classes, orders and even families, and supergroups tend to be partially eliminated. Often factors of these differences remain elusive, but organisms with mutualistic or parasitic lifestyles that are also common in soil animals and plants tend to evolve faster, as exemplified with the intracellular rhizarian parasite *Microcytos* (Hartikainen *et al*., 2014), or, living within terrestrial organisms, Microsporidia (Brinkmann *et al*., 2005).

The fast and heterogeneously evolving eukaryotic ribosomal genes also incorporate often introns of many hundreds of base pairs and supplementary loops that render the retrieval of certain sequences using modern HTS technology, such as Illumina's MiSeq, impossible. The presence of these insertions varies quickly in evolution, and closely related taxa can gain or lose them as illustrated in Heterolobosea (Geisen *et al*., 2015a), diatoms (Han *et al*., 2018) and in Arcellinid testate amoebae (Lara *et al*., 2008).

Among the two most commonly used variable regions of the 18S rRNA gene (v4 and v9), v4 is particularly prone to amplification biases (Vaulot *et al*., 2022). On the other hand, v4 is longer and provides more phylogenetic information (Fig. 2). The use of one region or the other depends of course on the research question, and experimental designs need to consider these limitations. In sum, there is no silver bullet solution to track all soil eukaryotic molecular diversity in metabarcoding studies. For eukaryotes, an homogenization of PCR protocols as recommended in the Earth Microbiome Project (Thompson, 2017) would systematically bias our vision of diversity against ‘non‐standard’ taxa. These do represent a substantial amount of the eukaryotic diversity, which might be largely lost in environmental eukaryotic DNA diversity studies.

## Future perspectives on eukaryotic metabarcoding in soils

**Fig. 4:**
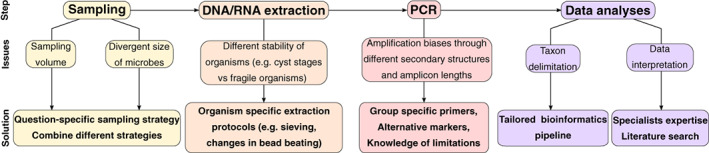
Illustration of the workflow followed in a eukaryotic metabarcoding experiment. Issues specific to eukaryotic metabarcoding are illustrated specifically, as well as solutions proposed to overcome these problems.

Despite the fact that eukaryotic metabarcoding approaches in soils are still in their infancy compared to prokaryotic studies, many methodological developments should overcome the current shortcomings. Here we list some potentials and recommendations to improve soil eukaryotic metabarcoding, which we summarize in Fig. 4:

1. Different diversity concepts need to be considered. While prokaryotic 16S rRNA OTUs can be considered as diversity proxies, in eukaryotes 18S rRNA OTUs correspond to real taxa that most of the time can be annotated to genus or family level or above, but rarely to species level. The level of taxonomic definition depends on the group considered and the targeted gene.
2. Sampling needs to be optimized when larger protists and metazoa are targeted by upscaling sample sizes or possibly concentrating organisms that cover also the local heterogeneity in organismal distribution. Holistic eukaryotic analyses covering protists and at least the most abundant ‘microbial sized’ animals (e.g. nematodes and rotifers) could be conducted by pooling nucleic acid extractions obtained from different protocols dimensioned for different organisms size classes. Including also the larger soil eukaryotes can be of fundamental value in ecological studies through their role as major microbiome predators, which influence its composition and functioning.
3. Environmental nucleic acids extraction and PCR will invariably bias the true information on eukaryotic diversity. Consensus approaches need to be applied by using the most generalist protocols (Santos *et al*., 2017; Vaulot *et al*., 2022), which allow cross‐sample comparisons. Still, it must be kept in mind that taxon‐specific protocols are needed for a substantial part of all soil eukaryotic clades if the entity of eukaryotic diversity is meant to be studied.

Next, we list a series of developments that will potentially open new research avenues in eukaryotic metabarcoding.

### Functional annotations of eukaryotic metabarcoding data are readily possible compared to bacterial metabarcoding data

A major opportunity offered by eukaryotic environmental DNA surveys is the possibility to infer functions from taxonomic barcoding sequences. Eukaryotes keep to a large extent the inherited functions, like for instance photosynthesis in diatoms or active phagocytosis in Amoebozoa. This characteristic, which derives from the stability of eukaryotic genomes, allows a relatively reliable functional annotation of OTUs (Singer *et al*., 2021). Functions are conserved along clades at different taxonomic depths. For instance, in some protist groups, like in ciliates, function is conserved within classes to a certain extent, and a finer taxonomical resolution that can be reached with 18S rRNA sequences may refine even more the functional assignment (Mieczan, 2009). Thus, annotation based on ribosomal sequences can be relatively precise (Lara *et al*., 2007; Lara and Acosta‐Mercado, 2012). Other groups can be more challenging to characterize, like chrysophytes, where photosynthetic capacity was lost many times during the groups evolutionary history (Boenigk *et al*., 2005). This characteristic makes functional inference based on ribosomal sequences difficult in this group known to include both phagotrophic and phototrophic organisms. This situation is probably going to change as more species will be functionally characterized in the future. Therefore, functional annotations based on relatively easily obtainable 18S rRNA gene reads can be of major additional benefit to normal taxonomic information as the ecological role of distinct and entire communities of eukaryotes can be studied. It is a matter of time to implement an automated functional annotation tool for all soil eukaryotes.

Bacteria can generally not be reliably assigned a function in soils. Although functions can be related to some OTUs in bacteria and archaea (e.g. cyanobacteria, methanogens, etc.), the immense often unknown diversity in soils along with the plasticity of their genomes renders this prediction perilous. Some bacterial functional prediction pipelines like FAPROTAX have been used with some success in soil (Sansupa *et al*., 2021). However, this approach is based on the assumption that if two organisms share the same 16S rRNA gene sequence, then their genome (and thus their function) should be identical; an assumption that does not take into account bacterial genomic plasticity. In this sense, an approach through direct sequencing of functional genes retrieved from environmental samples has been favoured for years (see e.g. Gremion *et al*., 2004). In addition, recent deep‐sequencing efforts that reconstitute full genomes from metagenomes, metagenome‐assembled genomes (MAGs), are starting to be applied and reveal reliable potential gene functions of soil bacteria (Crits‐Christoph *et al*., 2018). Yet, this approach is only possible for a few numerically dominant taxa as many tens of thousands of bacterial taxa constitute the immensely large metagenome of a fraction of a gram of soil. Therefore, most bacteria remain functionally unknown in soils. It must be noted that, even worse than for bacteria, MAGs of soil eukaryotes cannot be assembled. This is caused by larger genomes and eukaryotic rarity compared to bacteria and archaea (Xiong *et al*., 2021), which diminishes eukaryotic sequence information in metagenomic data. Furthermore, eukaryotic functional genes can often not even be assigned to known functions (Sibbald and Archibald, 2017). Yet, and until new developments of sequencing technologies allow increasing even more sequencing depth to the point that eukaryotic genomes are assembled from metagenomes, the possibility of characterizing eukaryotic functional diversity directly from metabarcodes provides great insights into terrestrial ecosystems functioning.

### The quantitative aspect of eukaryotic metabarcoding

Quantitative aspects are important information in any ecological study, and it would be desirable to include them in environmental metabarcoding surveys. Quantitative data such as abundance and especially biomass information are needed to estimate, for example, the importance of biodiversity in the global carbon, nitrogen and phosphorus cycles through food chains and across ecosystems. The question on how to interpret the number of reads obtained per OTU has been subject to a heated debate. Numbers of reads result approximately from the number of single cells times the number of copies per cell. These numbers vary between and within bacterial and archaeal taxa, spanning one order of magnitude, which prevents establishing a direct relationship between numbers of cells and numbers of reads (Stoddard *et al*., 2015). In eukaryotes, these numbers may vary by up to six orders of magnitude (Lavrinienko *et al*., 2021). As a consequence, studies show discrepancies between numbers of individuals and proportions of eukaryotic phylotypes in environmental metabarcoding studies (Gong and Marchetti, 2019). Ciliates, for instance, tend to be overrepresented, possibly due to their highly multiploid macronuclei (Geisen *et al*., 2015b). These numerical biases are combined with all potential flaws mentioned above (DNA extraction, PCR and sequencing). Therefore, Jurburg *et al*. (2021) even suggested to handle eukaryotic metabarcoding data as presence/absence, without any quantitative interpretation. This approach, however, needs to be very carefully considered for most ecological studies as numerical comparisons can provide vastly extended information compared to presence/absence data; in this sense, numerical data are far more important than richness information.

While copy numbers per genome do vary, total biovolumes of the organisms represented by different phylotypes can correlate with numbers of reads per OTU. Gonzalez‐de‐Salceda and Garcia‐Pichel (2021) recently found an allometric relationship, where cell biovolume and ribosomal operon copy numbers in microorganisms (including bacteria and archaea) are correlated well. A similar relationship exists also for multicellular organisms, namely, nematodes (Schenk *et al*., 2019). Correlating eukaryote biovolumes with numbers of reads in metabarcoding studies is in our view the way to bring quantitative aspects into the currently at best semi‐quantitative, qualitative metabarcoding data of soil eukaryotes. These biovolumes can be converted into C‐biomass equivalents, such as done for nematodes (van den Hoogen *et al*., 2019), which can be instrumental in following nutrient flows in ecosystems (Gilbert *et al*., 1998). However, calibration studies are needed to evaluate the biases inherent to a given eukaryotic taxon. A large database of individual species biovolumes is needed for broader use in ecological studies. In the meanwhile, sequence read numbers can still be considered as semi‐quantitative, but should be used with caution when applying numerically sensitive analyses as in correlation networks.

### Potential for species‐level resolution in eukaryotic metabarcoding

As stated above, the 18S rRNA gene often provides a rather coarse taxonomic resolution for most eukaryotic groups. However, other molecular markers can increase taxonomic resolution. Due to the closer comparability to animals and plants, a species‐level taxonomic resolution can be used to apply the ecological theoretical background developed for plants and animals during the past decades and centuries. For that purpose, several barcoding genes have been tested for their taxonomic definition in isolated organisms and on pure cultures. Primers have been designed in order to cover the largest possible range of organisms within their target groups.

While short‐read metabarcoding approaches have been standard since its implementation almost a decade ago to target soil eukaryotes (Bates *et al*., 2013), long‐read HTS approaches, such as PacBio and Nanopore, are rapidly developing. As their error rate per base pair is decreasing, they can potentially allow the retrieval of full‐length ribosomal operon, combining species or even population‐resolved taxonomic information based on the ITS region with the phylogenetic information provided by 18S and 28S rRNA genes (Jamy *et al*., 2020; Tedersoo *et al*., 2021).

Another promising gene for metabarcoding is the COI. This gene is conserved across most animals (Hebert *et al*., 2003) and is used for rotifers, where it largely outperforms the 18S rRNA gene in terms of taxonomic resolution (Fontaneto *et al*., 2019). COI is also a reference for soil microarthropods (Porter *et al*., 2019). Although priming regions are too variable to be used to scan all nematode diversity (Ahmed *et al*., 2019), COI is a promising marker to delimit species within nematode families (Bai *et al*., 2020). In soil protist metabarcoding, this marker was applied to Arcellinida (lobose testate amoebae) revealing niche differentiation between closely related species (Singer *et al*., 2018). Together, metabarcoding focusing on genes other than the 18s rRNA gene and long‐read sequencing provide ultra‐high taxonomic resolution, which can be used for in‐depth studies on taxonomic profiles, species‐level biogeographic analyses or bioindication/tracking invasive species.

## Concluding remarks

We here provide an overview of how the major differences that exist between prokaryotes and eukaryotes need to be taken into consideration when performing metabarcoding studies, from the conceptualization of experiments to data interpretation. Particularly in terrestrial systems, methods have first been optimized for prokaryotes, which leads to suboptimal experimental designs and misinterpretations of results in studies aiming at studying soil eukaryotes. Furthermore, we provide perspectives on ecologically relevant data that can be obtained through eukaryotic metabarcoding approaches, like functional information on soil eukaryotes. Methodological developments, thorough calibration and optimization efforts are still needed to refine quantitative interpretation of sequence read numbers; however, it can be foreseen that many methodological gaps will be filled in the years to come. Then, the possibility of applying fast evolving markers used for barcoding opens the door to many applications in academic (phylogeography, community ecology, etc.) and applied fields (bioindication, pathogen monitoring, etc.). Taken together, all these approaches will reveal the great power of eukaryotic environmental microbiology. Still, until then, it is of crucial importance to involve experts in studies; and, furthermore to train students in all steps of a given study, from design to interpretation.
